# Concomitant Intracardiac Shunt and Venous Collaterals After Fontan Procedure: A Case Report of Percutaneous Management

**DOI:** 10.3390/jcdd13060257

**Published:** 2026-06-10

**Authors:** Georgiana Pintea Bentea, Marielle Morissens, Pierre-Emmanuel Massart, Jose Castro Rodriguez

**Affiliations:** 1 Department of Cardiology, CHU UCL Namur Sainte Elisabeth, 5000 Namur, Belgium; 2 Department of Cardiology, CHU Brugmann, 1020 Brussels, Belgium

**Keywords:** congenital heart disease, vascular plug, intracardiac shunt, Fontan procedure

## Abstract

A 40-year-old man with complex congenital heart disease (double-inlet left ventricle with transposition of the great arteries), previously treated with a Blalock–Taussig shunt in infancy and a modified Fontan procedure (including superior vena cava-to-pulmonary artery anastomosis, atriopulmonary connection, and tricuspid valve closure with a Dacron patch), presented to the emergency department with worsening dyspnea and hypoxemia (SpO_2_ < 80%). Echocardiography suggested a shunt through the tricuspid patch, possibly related to prior atrial flutter ablation. Cardiac catheterization confirmed an approximately 10 mm fenestration in the calcified patch causing a significant bidirectional shunt, along with two fistulae between the innominate vein and the left atrium. The fenestration was successfully closed using a septal occluder via right femoral venous access under transesophageal echocardiographic guidance. The venous collaterals were occluded with vascular plugs via right femoral and left brachial approaches. Technical success of the closure of the intracardiac and the venous shunts was confirmed angiographically at the end of the procedure. Oxygen saturation improved immediately from 72% to 91% and remained stable at the 2-year follow-up. Similarly, NYHA functional class improved from IV to II and episodes of tachycardia became less frequent and better tolerated, with sustained benefit throughout follow-up.

## 1. Introduction

For many patients with a functionally univentricular heart, Fontan surgery represents the planned destination therapy. However, it can be associated with multiple complications, especially due to the increased systemic venous pressure.

## 2. Case Report

We present the case of a 40-year-old male patient born with a double-inlet left ventricle form of univentricular heart, in conjunction with transposition of the great arteries. He benefited shortly after birth from a Blalock–Taussig shunt and later on from a modified Fontan procedure [1], which entailed an anastomosis of the superior vena cava to the right pulmonary artery, an atriopulmonary connection, and occlusion of the right atrioventricular valve by a Dacron patch. His cardiac follow-up was marked during his adolescence by multiples episodes of atrial tachyarrhythmia requiring repeated cardioversions and atrial flutter ablation. Subsequently, he developed a right-to-left shunt via small holes in the Dacron right atrioventricular obstructive patch. The shunt remained stable for almost two decades.

**Figure 1:**
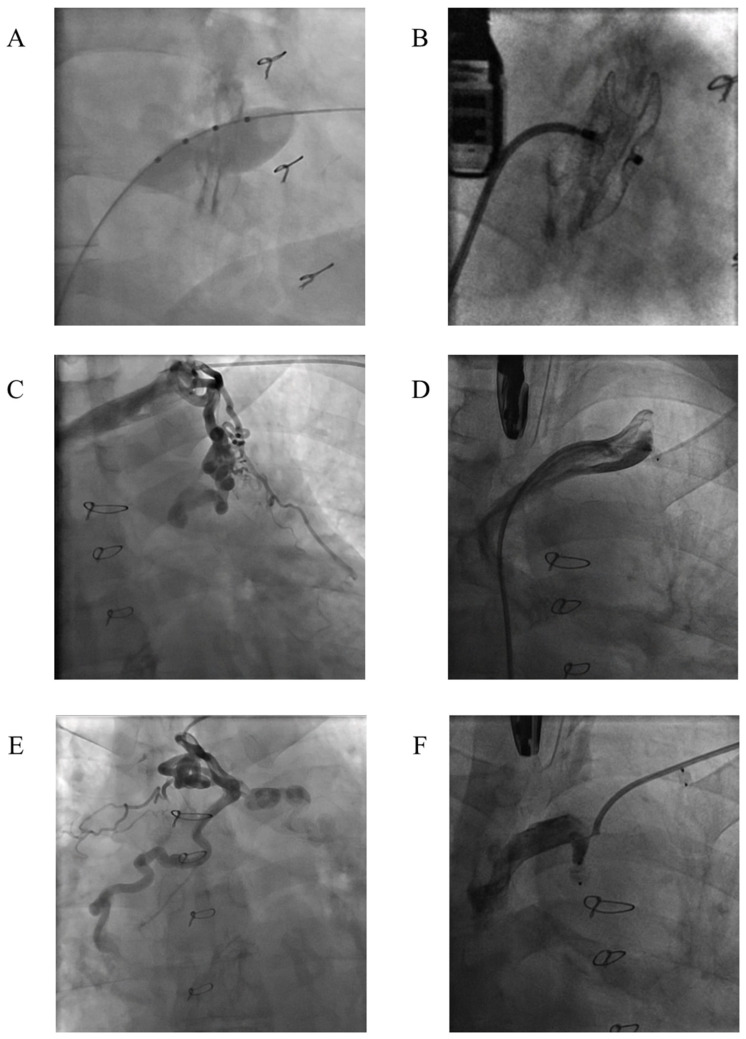
Percutaneous treatment of intracardiac shunt (**A**,**B**) and systemic-to-pulmonary venous collaterals (**C**–**F**) in the aftermath of a Fontan procedure.

Almost 20 years later, the patient presented to the emergency department with worsening dyspnea and deterioration of transcutaneous oxygen saturation on a home pulse oximeter (SpO_2_ < 80%), which were attributed to episodes of atrial tachyarrhythmias. However, the echocardiography showed a worsening of the shunt at the level of the tricuspid obstructive patch. Subsequently, the patient benefited from a cardiac catheterization that confirmed a fenestration of approximatively 10 mm through the middle of the calcified Dacron patch responsible of a significant and bidirectional shunt. A balloon occlusion test (Figure 1A) was performed at the level of this fenestration, which improved significantly the arterial saturation of oxygen from 72% to 92%, without deleterious hemodynamical consequences.

Furthermore, angiography identified two fistulae between the innominate vein and the left atrium, likely contributing to worsening cyanosis. Following Heart Team discussion and consensus, and based on favorable results of the balloon occlusion test, we proceeded with percutaneous closure of the fenestration in the Dacron tricuspid membrane. We decided, as well, to treat percutaneously the two venous shunts, as the Fontan circuit pressure was inferior to 18 mmHg, which was identified as a favorable prognostic factor previously described in the literature [2]. We proceeded to close the fenestration with an Amplatzer Septal Occluder 18 mm device under transesophageal echocardiography guidance via a right femoral vein approach (Figure 1B). The two systemic-to-pulmonary venous collaterals were closed with two Amplatzer Vascular Plug IIs of 4 and 6 mm, one via the right femoral vein (Figure 1C,D; Videos S1, S3, S4) and the more lateral one requiring a switch to a left brachial vein approach (Figure 1E,F; Videos S2, S5, S6). Technical success of the closure of the intracardiac and the venous shunts was confirmed angiographically at the end of the procedure. Furthermore, the patient presented an immediate improvement in arterial saturation of oxygen during the procedure (91% coming from 72% in the beginning of the procedure), which persisted at two-year follow-up. In parallel, the patient’s clinical status improved substantially, with NYHA functional class improving from IV to II. The patient not only tolerated episodes of tachycardia better, but also experienced them far less frequently, and these benefits were sustained throughout the follow-up period. There was no difference in single-ventricle ejection fraction (60% before and after), and no significant change in NT-proBNP levels (763 ng/L after vs. 797 ng/L before).

## 3. Discussion

The Fontan circulation is associated with substantial long-term morbidity due to chronically elevated systemic venous pressure and reduced cardiac output. As survival into adulthood has improved, a broad spectrum of late complications has become increasingly recognized, affecting the cardiovascular, pulmonary, hepatic, lymphatic, and thromboembolic systems [3].

Cardiovascular complications are among the most common and include ventricular dysfunction, atrioventricular valve regurgitation, arrhythmias and thromboembolic events. Atrial arrhythmias are particularly frequent in older atriopulmonary Fontan connections and contribute significantly to morbidity and hospitalization [3].

Cyanosis after Fontan palliation is frequently related to the development of veno-venous collaterals or pulmonary arteriovenous malformations. Veno-venous collaterals are common, with reported prevalence ranging from approximately 12% to as high as 30–60%, depending on the imaging modality and patient population studied. These collateral vessels create right-to-left shunting, leading to progressive desaturation, exercise intolerance, erythrocytosis, and an increased risk of paradoxical embolism. Intracardiac right-to-left shunts may also persist or develop due to residual fenestrations, baffle leaks, or septal defects [2].

The clinical outcome of veno-venous percutaneous closure described previously in the literature varies greatly [4,5,6].

The recent literature highlights transcatheter therapy as a key component in the management of Fontan-associated venous collaterals. Contemporary series and reviews confirm that percutaneous embolization is safe and effective, achieving improvement in oxygen saturation and symptoms in most patients, although recurrence or persistence of collaterals may occur and necessitate repeat interventions [7].

More recent interventional data also emphasize that veno-venous collaterals are part of a broader spectrum of Fontan pathway decompression mechanisms, often associated with elevated systemic venous pressures or Fontan failure physiology. Transcatheter occlusion using coils, vascular plugs, or other embolic devices is now widely considered first-line therapy in anatomically suitable cases, with high procedural success rates reported across contemporary single-center series and systematic reviews [7].

We describe a case of concomitant diagnosis of intracardiac shunt and venous collaterals in the aftermath of a Fontan procedure by cardiac catheterization and their combined percutaneous management during a single procedure with very good outcome at one-year follow-up. Adequate indication of such procedures and the management of congenital heart disease in specialized centers are essential.

The closure of an intracardiac shunt may indeed be poorly tolerated from a hemodynamic standpoint. However, this phenomenon is less frequently observed with veno-venous shunts, as excessively elevated venous pressures generally lead to the rapid recruitment of alternative collateral pathways. These collaterals correspond to pre-existing vascular channels rather than de novo angiogenesis. In the present case, the decision to concomitantly close both the intracardiac and veno-venous shunts was supported by the patient’s marked hemodynamic instability during episodes of tachycardia, which were associated with profound oxygen desaturation. Under these circumstances, the benefit–risk assessment was considered clearly favorable to complete closure of all shunts.

We hypothesize that the small perforations in the Dacron right atrioventricular obstructive patch may have been inadvertently created during cavotricuspid isthmus-dependent atrial flutter ablation.

This hypothesis is supported by the fact that the intracardiac shunt was first detected shortly after cavotricuspid isthmus ablation, whereas no shunt had been present on prior imaging. Although spontaneous degenerative changes in the patch cannot be excluded, the close temporal association suggests a possible procedure-related mechanism.

The cavotricuspid isthmus ablation was performed in the right atrium, in close proximity to the Dacron patch and within an electrically silent region. Radiofrequency energy was delivered at standard settings, with a power of approximately 40 W; however, this may have resulted in localized thermal injury to the adjacent patch material.

To our knowledge, there are no previous publications describing this complication.

Initially, the worsening of cyanosis of the patient was inaccurately attributed to paroxysms of atrial tachyarrhythmias. However, the patient continued to present paroxysms of atrial tachyarrhythmias after the percutaneous treatments of the shunts, without any impact on the dyspnea or arterial oxygenation. We could hypothesize that previously the episodes of atrial tachyarrhythmias would induce desaturation by reducing the systemic cardiac output, hence reverting the shunt right to left. This observation highlights the pathophysiological complexity of congenital heart disease and the need for specialized centers for their management.

As previously mentioned, Fontan surgery represents a palliative strategy for patients with single-ventricle physiology and may be associated with multiple long-term complications, particularly related to chronically elevated systemic venous pressure.

Here, we report the unusual case of a patient diagnosed nearly 40 years after Fontan surgery with the rare coexistence of an intracardiac right-to-left shunt and extensive venous collaterals, both identified by interventional cardiac catheterization and successfully treated through a percutaneous approach. In this case, the originality lies in the concomitant closure of both intracardiac and veno-venous shunts, justified by the patient’s severe hemodynamic instability during tachycardia episodes, associated with profound desaturation, resulting in a clearly favorable benefit–risk balance for complete shunt occlusion.

We hypothesize that the intracardiac shunt resulted from the development of small perforations in the Dacron tricuspid occlusion patch following cavotricuspid isthmus ablation for atrial flutter, representing a highly uncommon and previously scarcely described mechanism of late cyanosis after Fontan palliation.

This case also highlights the importance of comprehensive hemodynamic and anatomical evaluation in Fontan patients presenting with worsening cyanosis. In our patient, desaturation had initially been attributed to recurrent atrial tachyarrhythmias, whereas cardiac catheterization ultimately revealed significant venous collaterals and an intracardiac shunt amenable to transcatheter treatment.

## Data Availability

The data presented in this study are available on request from the corresponding author due to privacy reasons.

## Supplementary Materials

The following supporting information can be downloaded at: https://www.mdpi.com/article/10.3390/jcdd13060257/s1.
